# Supplementary material for: The functional interactome landscape of the human histone deacetylase family
Source: Mol Syst Biol. 2013 Jun 11;9:672. doi: 10.1038/msb.2013.26 (PMC3964310; doi:10.1038/msb.2013.26)
Supplement: Supplementary Figures and Tables [file msb201326-s1.pdf]

Supplementary Information Table of Contents

Joshi et al., "The functional interactome landscape of the human histone deacetylase family"

	Description	Pg
Supplementary Figure S1	Localization of EGFP and EGFP-tagged HDACs in CEM-T and HEK293 cells	2
Supplementary Figure S2	Localization of endogenous HDAC11 in wild-type CEM-T cells	3
Supplementary Figure S3	ROC plots guide selection of SAINT score filters	4
Supplementary Figure S4	Shared interactions between HDAC1-10 and HDAC11	5
Supplementary Figure S5	STRING functional networks from HDAC11 protein associations	6
Supplementary Figure S6	Cellular functions of HDAC11 and its interactions	7
Supplementary Figure S7	Correlation of protein abundance and IDIRT relative stability	8
Supplementary Figure S8	Validation of HDAC1 interactions by reciprocal isolation	9
Legends	Supplementary Table Legends	10
Supplementary Table S1	Optimized lysis buffer conditions for immunoaffinity purifications of HDACs and co-isolating proteins from HDAC-EGFP CEM-T cell lines	11
Supplementary Table S2	Excel File: Specific HDAC interactions from SAINT analysis	NA
Supplementary Table S3	Excel File: Non-specific proteins	NA
Supplementary Table S4	Excel File: HDAC11 associations from STRING Network	NA
Supplementary Table S5	Excel File: SAINT and I-DIRT comparative analyses	NA
Supplementary Table S6	Primers used in construction of HDAC cell lines	12
Supplementary Table S7	Primers used in QuikChange mutagenesis for HDAC2	13
Supplementary Table S8	Primers used to assess splicing defects following siRNA-mediated HDAC11 knockdown (see Figure 5E)	14

A

B

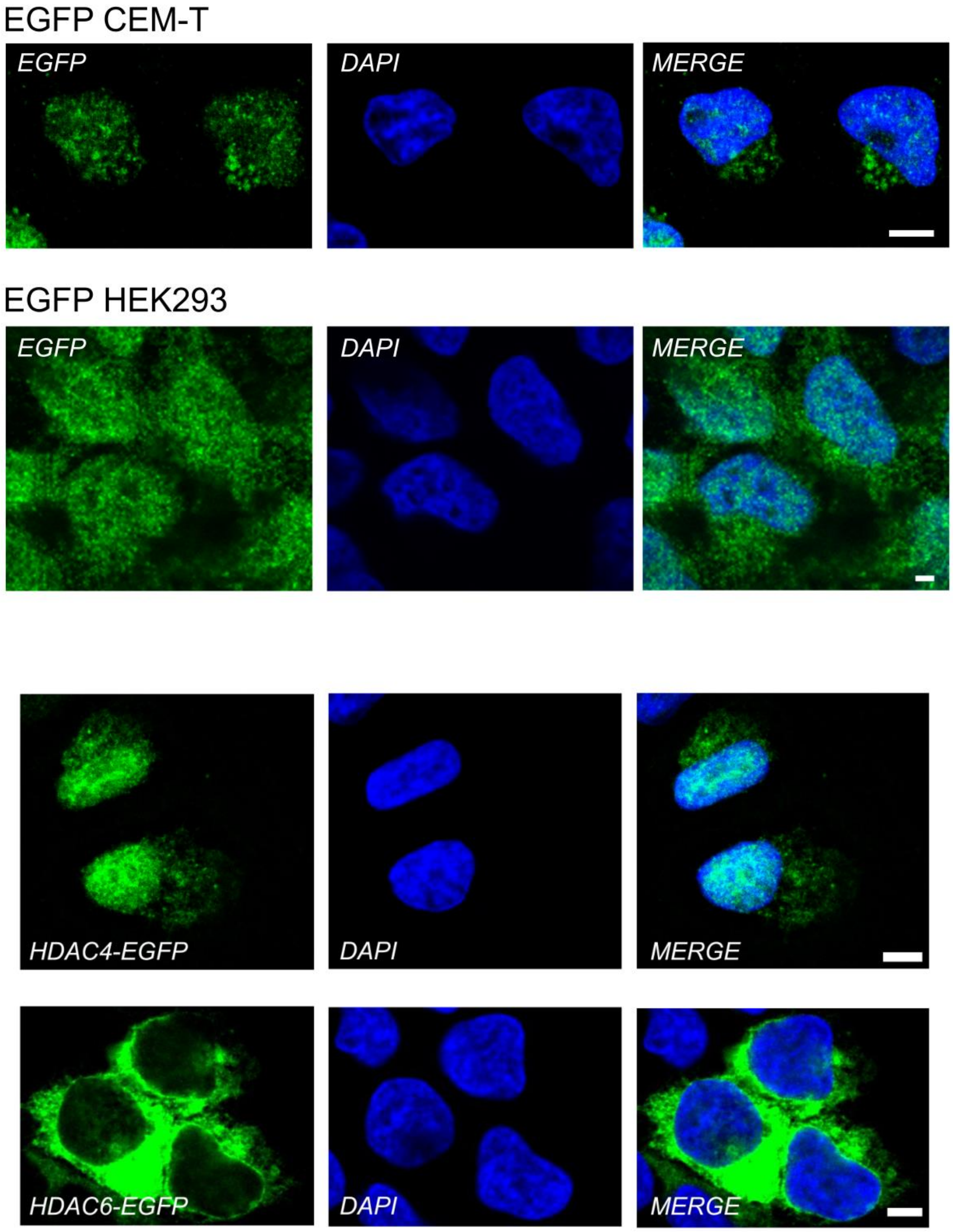

Supplementary Figure S1. Localization of EGFP and EGFP-tagged HDACs in CEM-T and HEK293 cells.

(A) CEM-T and HEK293 cell lines stably expressing EGFP. Scale bar = 5 µm. (B) CEM-T cell lines stably expressing HDAC4-EGFP or HDAC6-EGFP. EGFP (green); nucleus (blue, DAPI). Scale bar = 5 µm. Images acquired using either a Leica SP5 (with a 63X oil immersion lens) or a NikonA1 (with a 100X oil immersion lens) confocal microscope.

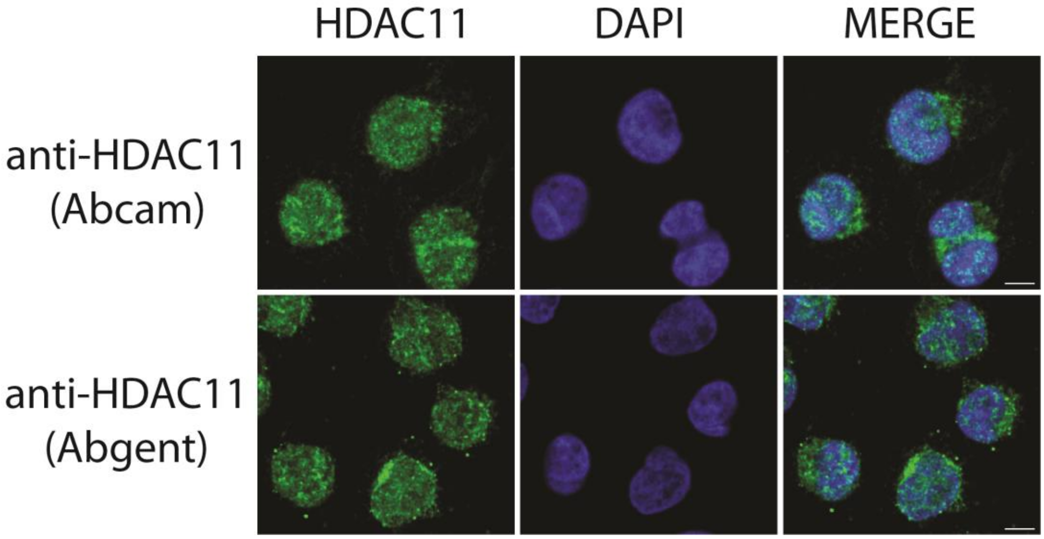

Supplementary Figure S2. Localization of endogenous HDAC11 in wild-type CEM-T cells.

Localization of endogenous HDAC11 in CEM-T cells using anti-HDAC11 antibody (green); two different antibodies were used as indicated (top and bottom panels); nuclear staining is indicated by DAPI (blue). All images were taken on a Leica SP5 confocal microscope with a 63X oil immersion lens; Bar 5 μm.

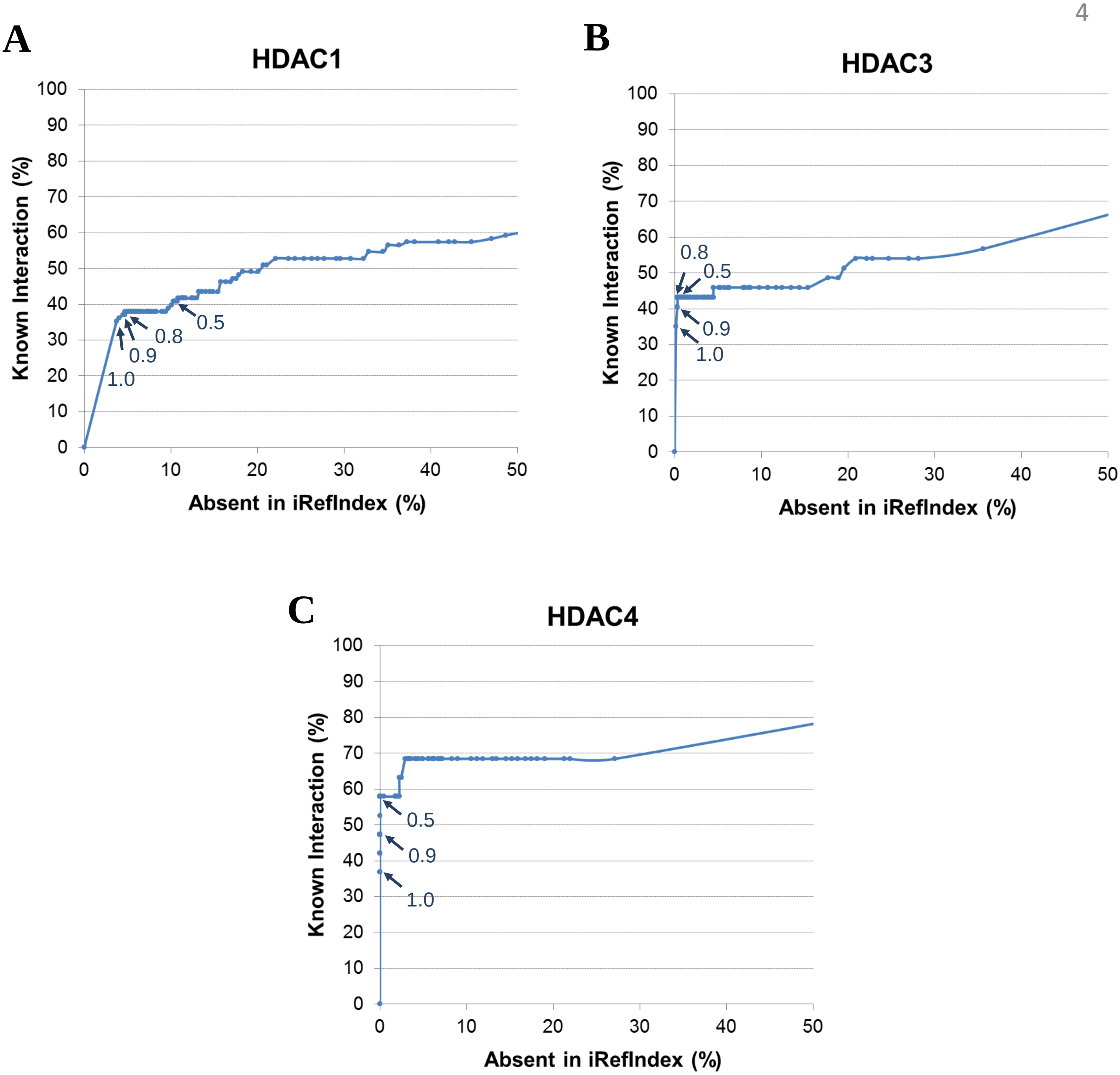

Supplementary Figure S3. "ROC" plots guide selection of SAINT score filters.

Shown above are "ROC" plots for (A) HDAC1, (B) HDAC3, and (C) HDAC4, which compares % of known interactions versus % not present in iRefIndex database as a function of SAINT scores (labeled arrows). All identified proteins within each immunoisolation were scored as either a positive hit (known interaction based on its presence in the iRefIndex interaction database (2012-12, ver. 9) and manual literature annotation) or as a negative hit (not present in the iRefIndex database). This relationship derives from the classical ROC curve comparing true positive versus false positives rates (TPR vs FPR). Yet these values can not be accurately determined for most protein interaction datasets and so "ROC" plots were interpreted conservatively.

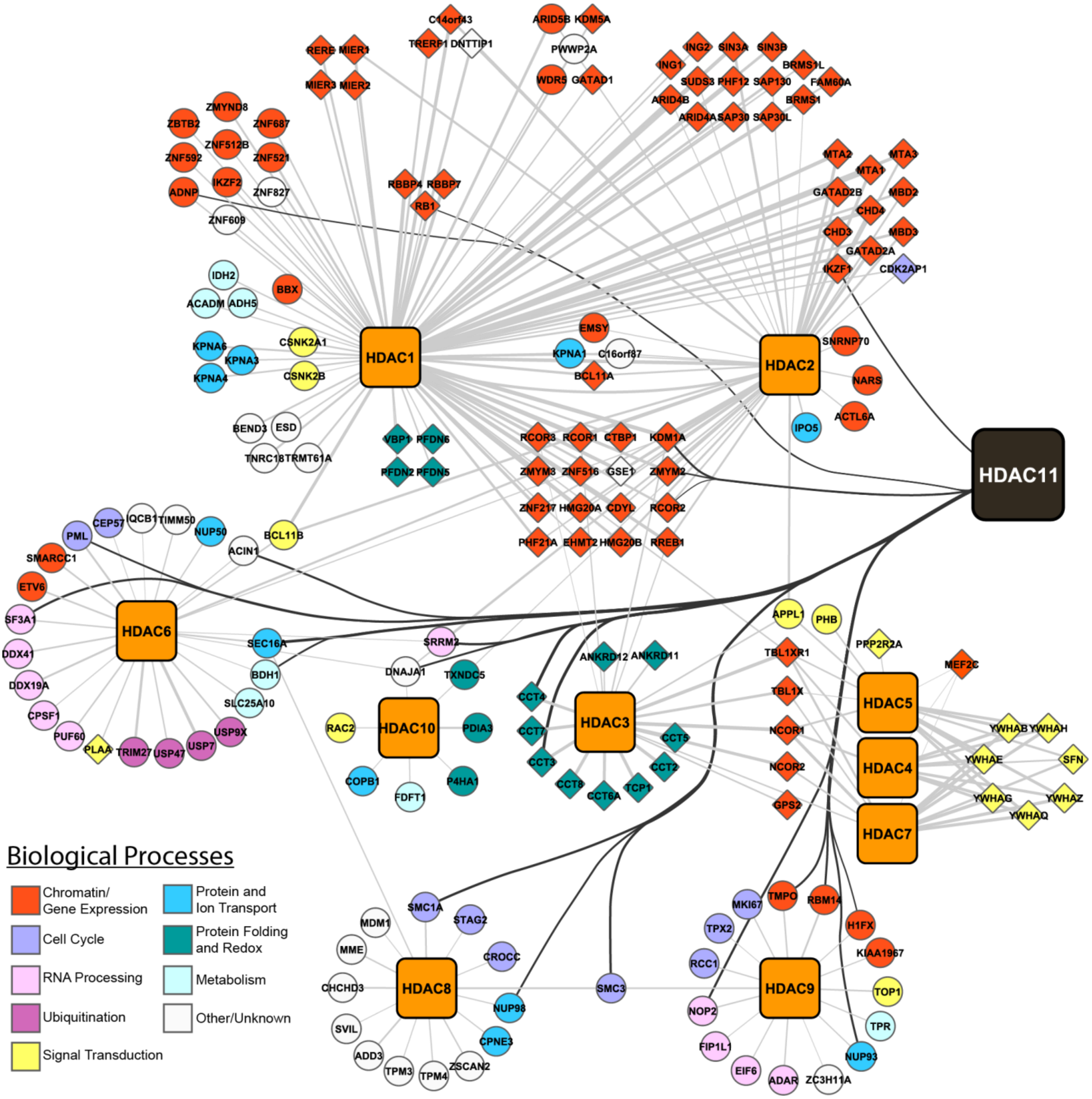

Supplementary Figure S4. Common interactions between HDAC11 and HDAC1-10. The common (intersection of) interactions between HDAC11 (Fig. 5A) and HDAC1-10 (Fig. 4) are illustrated by curved, dark grey edges. Edge thickness indicates $\log_2$-transformed prey spectral counts. Preys were manually classified and colored-coded by biological processes. Diamond and circle nodes indicate previously identified and uncharacterized HDAC interactions, respectively.

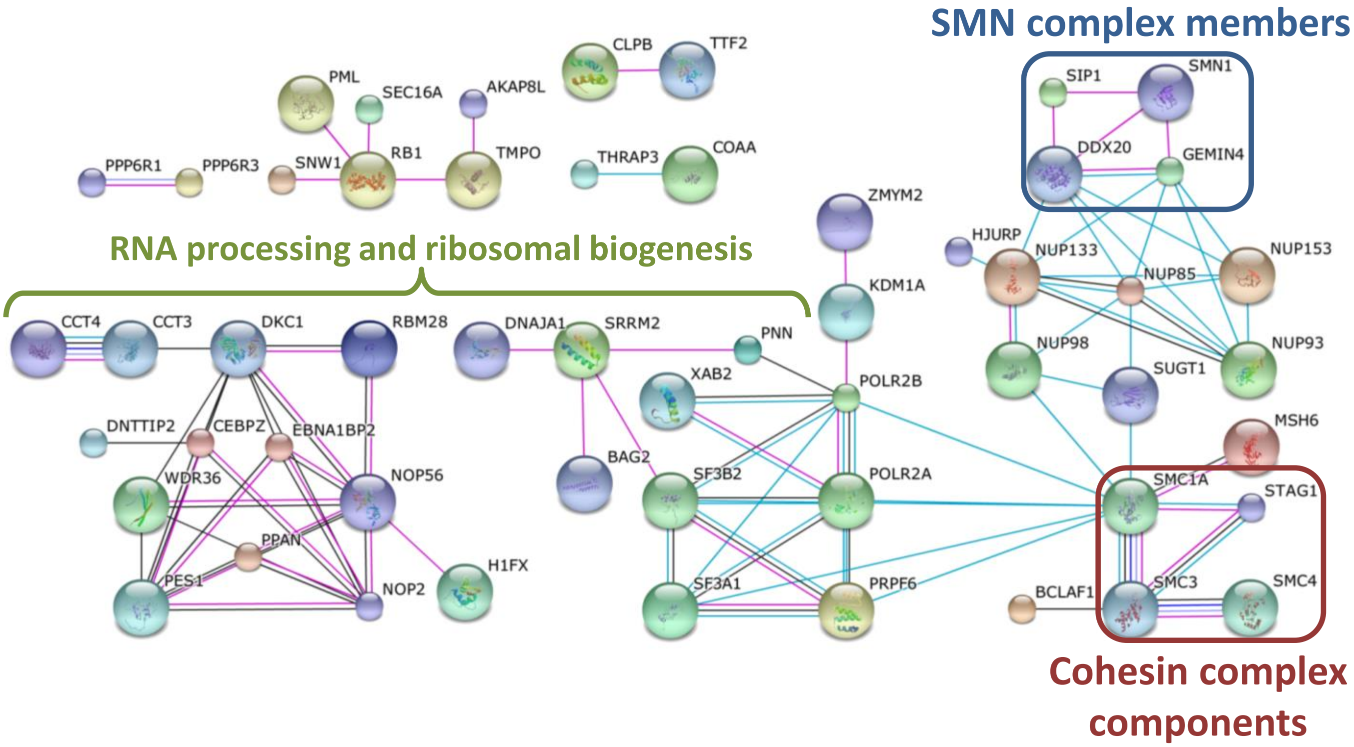

Supplementary Figure S5. STRING functional networks assembled from HDAC11 protein associations.

STRING analysis (ver. 9.0) was performed on the 124 HDAC11 candidate interactions (see Supplementary Table 2). The analysis resulted in functional connectivity among 55 proteins (shown above, STRING score > 0.50). Proteins without functional connectivity were omitted. The colors of the interaction edges represent previous evidence of association, either known or predicted: experiments (purple), databases (turquoise), homology (grey), and co-expression (black). Members of the Cohesin complex (blue box), SMN complex (red box), and proteins involved in RNA processing and ribosomal biogenesis (green bracket) are highlighted. If a protein structure or partial structure is known, it is indicated inside a large sphere, while proteins with unknown structures are represented as smaller spheres.

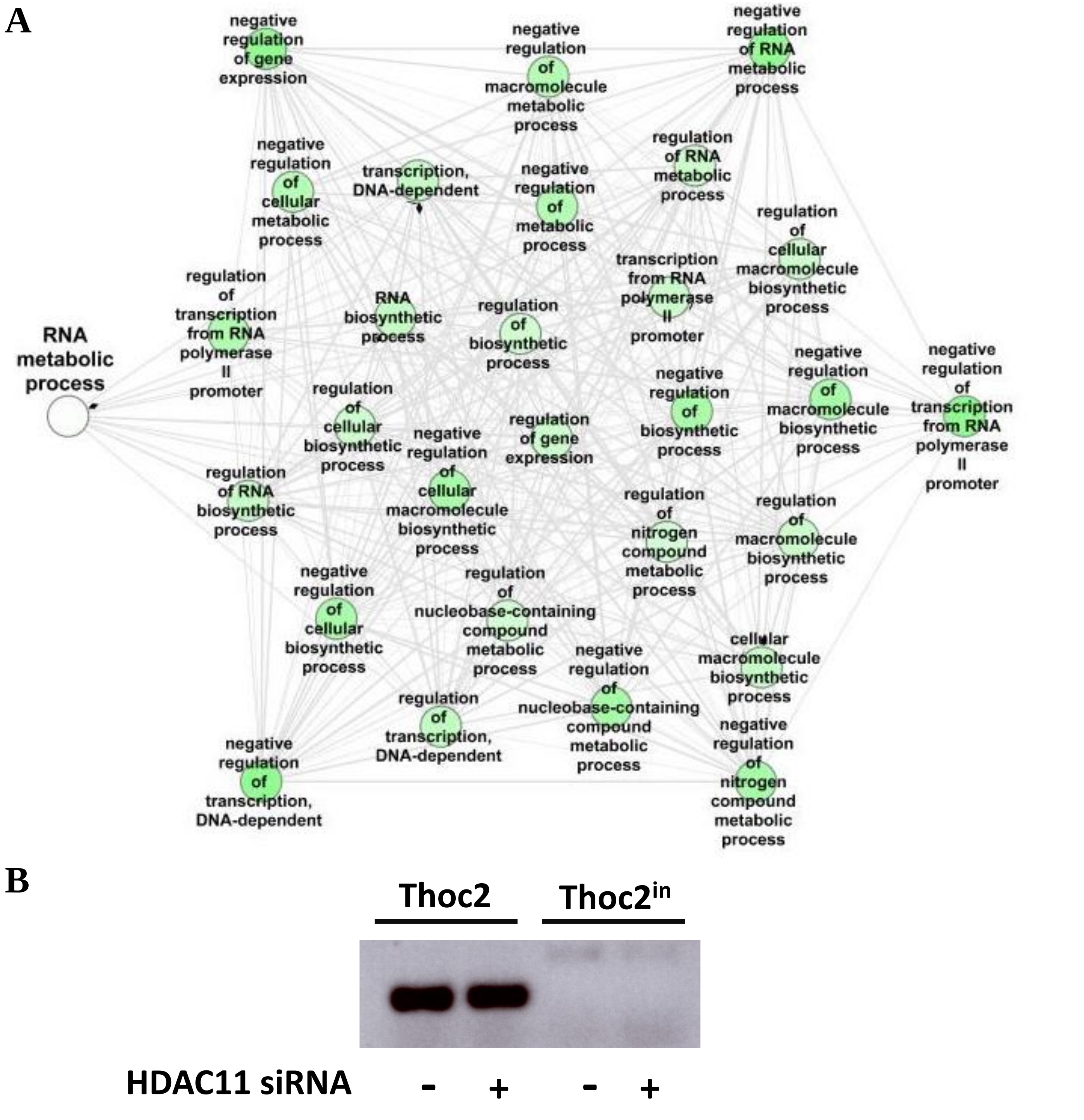

Supplementary Figure S6. Cellular functions of HDAC11 and its interactions.

(A) Complete node annotation of RNA metabolic process-related GO terms from Fig 5B. (B) Effect on splicing of ThoC mRNA following HDAC11 knockdown in WT CEM-T cells. As in Fig 5E, RNA levels upon treatment with HDAC11 siRNA (+) or a scrambled control (-) were measured by qRT-PCR and agarose gel electrophoresis was used to visualize the levels of indicated PCR products (n = 3). A representative agarose gel is shown for the Thoc2 gene and its mis-spliced product (Thoc2^{in}) containing the U12-type intron (I37).

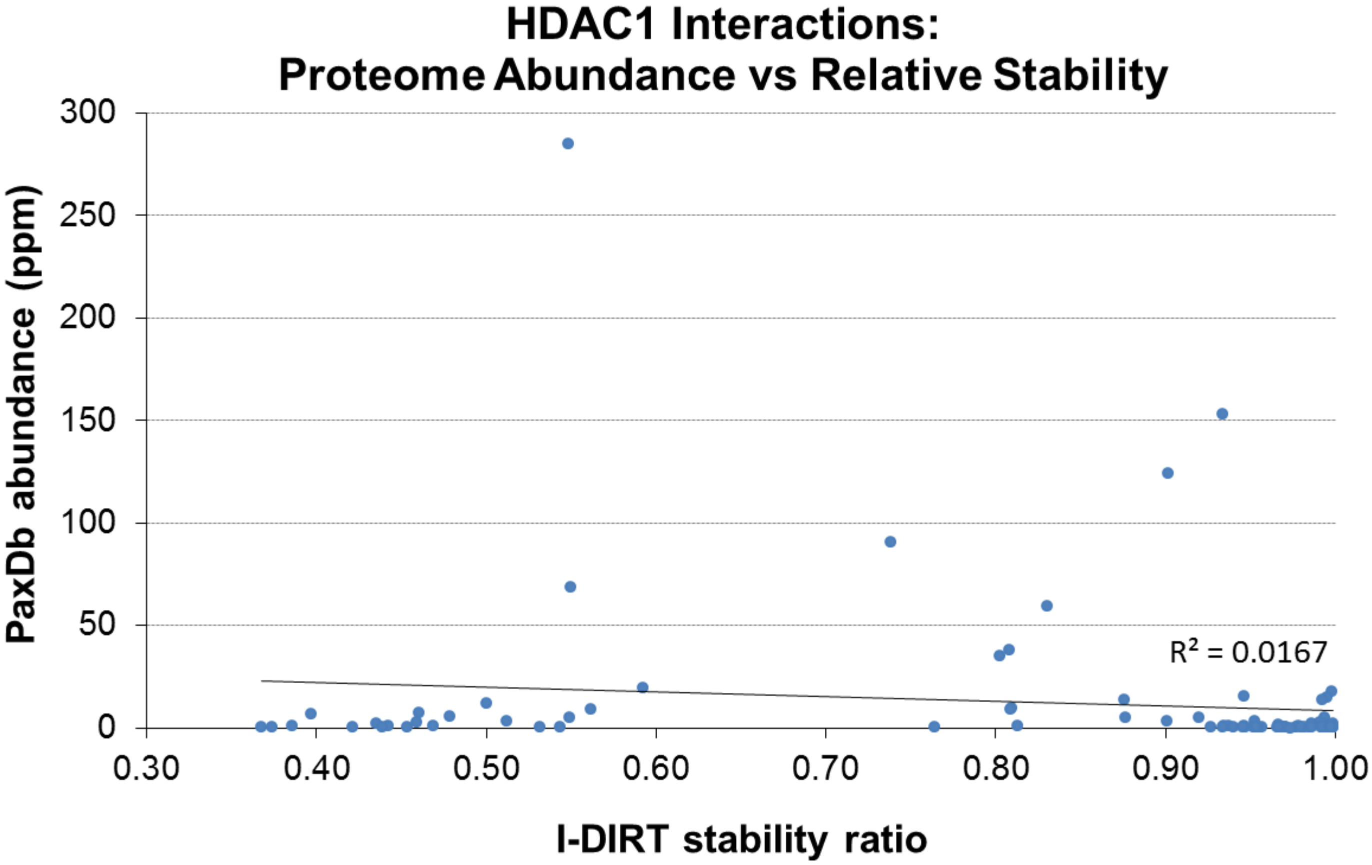

Supplementary Fig S7. Correlation of protein abundance and IDIRT relative stability.

Average proteome abundance compared to I-DIRT stability ratios for HDAC1 interactions (N = 89). I-DIRT stability ratios were from HDAC1 interactions in Fig 6D-E. Corresponding "proteome abundance" values based on large-scale spectral counting proteomic experiments were obtained from the human integrated dataset of the PAX database (www.pax-db.org). Linear regression line showing weak correlation between proteome abundance and relative stability.

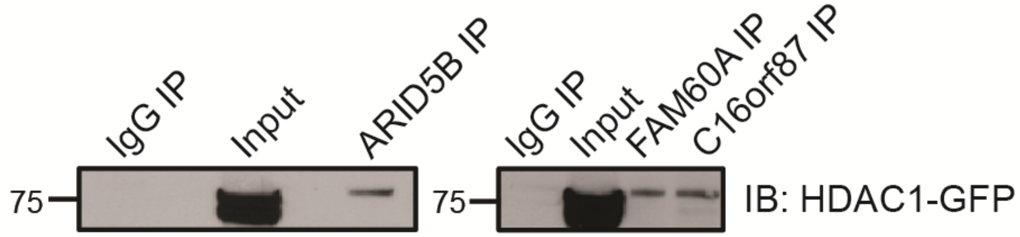

Supplementary Figure S8. Validation of interaction partners with HDAC1.

Reciprocal immunoaffinity purifications (IP) for HDAC1-GFP using antibodies against endogenous ARID5B, FAM60A, C16orf87. Immunoaffinity purification with IgG was used as a negative control. Immunoblotting with the anti-GFP antibody shows presence of the HDAC1-GFP in the corresponding IP. Input represents 2.5% of the total cell lysate.

Supplemental Excel Table Legends

Supplementary Table S2. Specific HDAC interactions from SAINT analysis. Protein interactions that were classified as specific after SAINT analysis. Separate sheets are provided for HDACs 1 – 10 and HDAC11. For each protein, the following information is provided: UniProt accession, gene symbol, description, protein length in amino acids, average SAINT score (Prob, HD1-10; iProb, HD11) and spectral counts (SC) for each HDAC IP and GFP IP controls (# 1- 7).

Supplementary Table S3. Excel spreadsheet of Non-specific proteins. Proteins that were classified as non-specific after SAINT analysis. Separate sheets are provided for HDACs 1 – 10 and HDAC11. For each protein, the following information is provided: UniProt accession, gene symbol, description, protein length in amino acids, average SAINT score (Prob, HD1-10; iProb, HD11) and spectral counts (SC) for each HDAC IP and GFP IP controls (# 1- 7).

Supplementary Table S4. Excel spreadsheet of HDAC11 interactions from STRING network. List of proteins having at least one known or predicted functional association among the HDAC11 candidate interactions (see Supplementary Table 2). Functional associations were predicted using the STRING database (ver. 9.0) as depicted in Supplementary Figure 2 (pg 3). For each protein, the following information is provided: UniProt accession, gene symbol, description, protein length in amino acids, spectral counts (SC) from each biological replicate, average spectral counts (AVG SC), cellular localization, biological process, average spectral counts normalized to protein length (NSc), normalized spectral abundance factor (NSAF), NSAF / proteome abundance (PAX) (NSAF/PAX), and normalized NSAF/PAX expressed relative to SUGT1 protein (normNSAF/PAX).

Supplementary Table S5. Excel spreadsheet of SAINT and I-DIRT comparative analyses. List of proteins that were identified in both SAINT and I-DIRT proteomic workflows. Separate sheets are included for HDAC1, HDAC3, HDAC5, and HDAC7 experiments. Proteins are listed in the following order: specific in both SAINT/IDIRT, specific in SAINT only, specific in I-DIRT only, and non-specific. For each protein, the following information is provided: UniProt accession, gene symbol, description, protein length in amino acids, average SAINT score, I-DIRT ratio. Evidence for previously reported interactions is indicated for databases (BioGRID, MINT, HPRD, DIP, Intact) or manual curation from literature (Y).

CELL LINE	BUFFER COMPOSITION
EGFP	TBT, 250mM NaCl, 0.5% Triton X-100, 1/100 (v/v) Protease Inhibitor Cocktail, 10 µg/ml DNAse
HDAC1-EGFP	TBT, 250mM NaCl, 0.5% Triton X-100, 1/100 (v/v) Protease Inhibitor Cocktail, 10 µg/ml DNAse
HDAC2-EGFP	TBT, 250mM NaCl, 0.5% Triton X-100, 1/100 (v/v) Protease Inhibitor Cocktail, 10 µg/ml DNAse
HDAC3-EGFP	TBT, 250mM NaCl, 0.5% Triton X-100, 1/100 (v/v) Protease Inhibitor Cocktail, 10 µg/ml DNAse
HDAC4-EGFP	TBT, 200mM NaCl, 1% Triton X-100,0.5% Sodium Deoxycholate, 1/100 (v/v) Protease Inhibitor Cocktail, 1/100 (v/v) Phosphatase Inhibitor Cocktail 2 (PhInC2), 1/100 (v/v) PhInC3, 4 µg/ml DNAse
HDAC5-EGFP	TBT, 250mM NaCl, 0.5% Triton X-100, 1/100 (v/v) Protease Inhibitor Cocktail, 1/100 (v/v) PhInC2, 1/100 (v/v) PhInC3, 4 µg/ml DNAse
HDAC6-EGFP	TBT, 250mM NaCl, 0.5% Triton X-100, 1/100 (v/v) Protease Inhibitor Cocktail, 10 µg/ml DNAse
HDAC7-EGFP	TBT, 250mM NaCl, 0.5% Triton X-100, 1/100 (v/v) Protease Inhibitor Cocktail, 10 µg/ml DNAse
HDAC8-EGFP	TBT, 250mM NaCl, 0.5% Triton X-100, 1/100 (v/v) Protease Inhibitor Cocktail, 10 µg/ml DNAse
HDAC9-EGFP	TBT, 150mM NaCl, 0.3% Triton X-100, 1/100 (v/v) Protease Inhibitor Cocktail, 1/100 (v/v) PhInC2, 1/100 (v/v) PhInC3, 4 µg/ml DNAse
HDAC10-EGFP	TBT, 250mM NaCl, 0.5% Triton X-100, 1/100 (v/v) Protease Inhibitor Cocktail, 10 µg/ml DNAse
HDAC11-EGFP	TBT, 200mM NaCl, 0.4% Triton X-100, 1/100 (v/v) Protease Inhibitor Cocktail, 10 µg/ml DNAse

Supplementary Table S1. Optimized lysis buffer conditions for immunoaffinity purifications of HDACs and co-isolating proteins from HDAC-EGFP CEM-T cell lines.

PRIMER	SEQUENCE
HDAC1	5’AAAA CTCGAG ATGGCGCAGACGCAGGGC<br>5’AAA GGATCC GGCCAACTTGACCTCCTC
HDAC2	5’AAA CTCGAG ATGGCGTACAGTCAAGGAG<br>5’AAA GGATCC GGGGTTGCTGAGCTGTTC
HDAC3	5’ AAA GAATTC ATGGCCAAGACCGTGGCC<br>5’ AAA GGATCC AATCTCCACATCGCTTTCCT
HDAC4	5’ AAA GAATTC ATGAGCTCCCAAAGCCATCC<br>5’ AAA AGATCT CAGGGGCGGCTCCTCTTC
HDAC5	5’ AAA CTCGAG ATGAACTCTCCCAACGAGTC<br>5’AAA AGATCT CAGGGCAGGCTCCTGCTC
HDAC6	5’AAA GTCGAC ATGACCTCAACCGGCCAG<br>5’ AAA AGATCT GTGTGGGTGGGGCATAT
HDAC7	5’AAA GAATTC ATGCACAGCCCCGGCGCT<br>5’AAA GCC ACC AGATCT GAGATTCATAGGTTCTTCC
HDAC8	5’ AAA GAATTC ATGGAGGAGCCGGAGGAAC<br>5’ AAA CTCGAG G GACCACATGCTTCAGATTCC
HDAC9	5’ AAA CTCGAG ATGCACAGTATGATCAGCTC<br>5’ AAA GCC ACC GGATCC<br>AGAGAACTTTAAAGACATACTT
HDAC10	5’ AAA GAATTC ATGGGGACCGCGCTTGTG<br>5’AAA CTCGAG G AGCCACCAGGTGAGGATG
HDAC11	5’ AAA GCC GAATTC ATGCTACACACAACCCAGCT<br>5’AAA GGATCC GGGCACTGCAGGGGGAAG
EGFP-FLAG	5’ AAA GGATCC G GCGGCCGC<br>ATGGTGAGCAAGGGCGAG<br>5’AAA AGATCT TCA CTTATCGTCGTCATCCTTGTAATC<br>GCGGCCGCCTTGTACAGCTCGTCCATG

Supplementary Table S6. Primers used in construction of HDAC-EGFP cell lines.

PRIMER	SEQUENCE
HDAC2 C167Y	5'GTG CTT GCC ATC CTT GAA TTA CTA AAG TAT CAT CAG AGA GTC<br>5'GAC TCT CTG ATG ATA CTT TAG TAA TTC AAG GAT GGC AAG CAC
HDAC2P477T.A480T	5'CAG ATA CCA AAG GAA CCA AAT CAG AAC AGC TCA GCA ACC C<br>5'GGG TTG CTG AGC TGT TCT GAT TTG GTT CCT TTG GTA TCT G

Supplementary Table S7. Primers used for Quick change mutagenesis for HDAC2.

PRIMER	SEQUENCE
GAPDH	5’ CGACAGTCAGCCGCATCTTCTTT<br>5’ GGCAACAATATCCACTTTACCAGAG
HDAC11	5’ TCTTCCTCCCCAACTTCCTT<br>5’ CTCCACACGCTCAAACAGAA
U2	5’ TTTGGCTAAGATCAAGTGTAGTATCTGTTC<br>5’ AATCCATTTAATATATTGTCCTCGGATAGA
U12	5’AACTTATGAGTAAGGAAAATAACGATTCG<br>5’CGACCTTTACCCGCTCAAAA
U4	5’ GCGCGATTATTGCTAATTGAAA<br>5’ AAAAATTGCCAATGCCGACTA
U4atac	5’ GCGCATAGTGAGGGCAGTACT<br>5’ GCACCAAAATAAAGCAAAAGCTCTA
ATXN10	5’ GTAAATGAGCTGGATGGTATCCCGTTG<br>5’ TGGCTTAGGGGTGTCTCTAGTAGATTTC
ATXN10_INTRON	5’ GTAAATGAGCTGGATGGTATCCCGTTG<br>5’ CCGGTCCCTGGTTAGTCACCACTGTAC
THOC2	5’ TGGGGGTTTCAAAACATAAAAGTGAAAGTC<br>5’ TCATTATCTGTGCTTGTCCGAGGACTTATGA
THOC2_intron	5’ TGGGGGTTTCAAAACATAAAAGTGAAAGTC<br>5’ CTTCCTCTCTGTGATAATATAACCTGAAAG

Supplementary Table S8. Primers used to assess splicing defects following siRNA-mediated HDAC11 knockdown.
